# Clinical and Economic Burden Associated With Anti-Cytomegalovirus (CMV) Prophylaxis Therapies in Adult Kidney Transplant Recipients (LECOCYT): An Observational Study

**DOI:** 10.3389/ti.2025.14342

**Published:** 2025-05-19

**Authors:** Nassim Kamar, Hannah Kaminski, Christophe Masset, Claire Castagné, Guilhem Tournaire, Xavier Bourge, Lionel Bensimon, Moustafa Naja, Stéphanie Degroote, Isabelle Durand-Zaleski, Christophe Legendre

**Affiliations:** ^1^ Department of Nephrology and Organ Transplantation, Toulouse Rangueil University Hospital, Institut national de la santé et de la recherche médicale - Unité Mixte de Recherche 1291 (INSERM UMR 1291), Toulouse Institute for Infectious and Inflammatory Diseases (Infinity), University Paul Sabatier, Toulouse, France; ^2^ Department of Nephrology-Transplantation-Dialysis-Apheresis, University Hospital of Bordeaux, Bordeaux, France; ^3^ Department of Nephrology, Institut de Transplantation Urologie-Nephrologie (ITUN), Nantes University Hospital, Nantes, France; ^4^ MSD France, Puteaux, France; ^5^ ClinSearch, Malakoff, France; ^6^ Unité de Recherche Clinique en Économie de la Santé d’Ile de France (URC-Eco), Assistance Publique-Hôpitaux de Paris (AP-HP), Université Paris Est Créteil, Créteil, France; ^7^ Nephrology and Renal Transplantation Department, Necker Hospital, Paris, France

**Keywords:** cytomegalovirus prophylaxis, economics, ganciclovir, valganciclovir, kidney transplant

## Abstract

The incidence of leukopenia and neutropenia associated with cytomegalovirus (CMV) prophylaxis in kidney transplant (KT) recipients is not well established. LECOCYT, a prospective observational multicenter study, aimed to investigate the clinical and economic burdens of CMV prophylaxis during the first 6 months post-transplantation. Grade 3 or 4 leukopenia or neutropenia was assessed in CMV-seropositive donors/CMV-seronegative recipients (D+/R-) who received current anti-CMV prophylaxis, and in CMV-seronegative donors/CMV-seronegative recipients (D-/R-) who did not. The economic burden in D+/R- was also evaluated. The adjusted odds ratio for grade 3 or 4 leukopenia or neutropenia was 5.16 [95% confidence interval: 1.97–13.53] for D+/R- group. The median costs, excluding the KT procedure, for D+/R- subgroup patients who experienced at least one episode of severe leukopenia or neutropenia were approximately €4,500 (Q1 = €561; Q3 = €10,000). D+/R- patients with no episode incurred significantly lower costs, with a median of nearly €2,100 (Q1 = €182; Q3 = €6,500) (p = 0.02). D+/R- patients with severe leukopenia or neutropenia had a higher rate of outpatient consultations than those without episode (73.9% vs. 57.6%, p = 0.002), and a higher average number of consultations per patient (5.5 ± 4.1 vs. 4.5 ± 3.3, p = 0.042) than D+/R- patients without. Anti-CMV prophylaxis in D+/R- transplant recipients was significantly associated with a higher rate of severe leukopenia or neutropenia compared to no prophylaxis in D-/R- recipients.

## Graphical Abstract

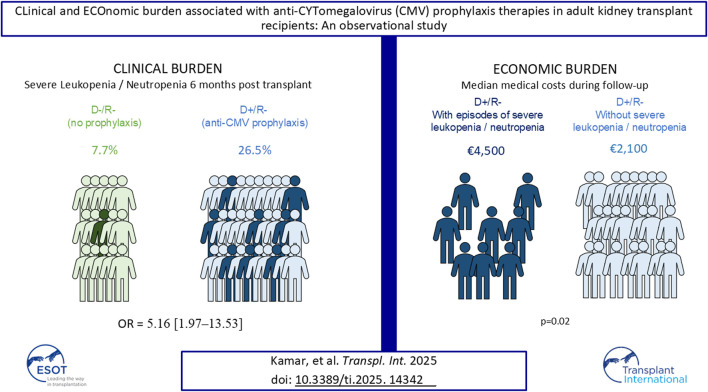

## Introduction

The risk of cytomegalovirus (CMV) infection in kidney-transplant (KT) patients is driven by the serostatus of the donor and recipient. The highest risk is for seronegative recipients (R-) receiving organs from seropositive donors (D+), followed by seropositive recipients (R+). The lowest risk is for seronegative donor-recipient pairs (D-/R-) [1].

To prevent CMV infection, two main strategies are employed: prophylactic and preemptive therapies. Prophylactic therapy involves administering antivirals shortly after transplantation, typically for three to 6 months, and is recommended for high-risk (D+/R-) and intermediate-risk (R+) patients [2]. Preemptive therapy requires regular monitoring of CMV viral load in the blood and starting antiviral treatment when a specific threshold is reached, ideally before symptoms appear [2]. The choice between prophylactic and preemptive strategies can vary widely among countries due to differences in healthcare policies and guidelines, availability and cost of antiviral drugs and CMV monitoring tests [3, 4]. A 2022 survey from the European Society for Organ Transplantation revealed that 95% of participating centers give an anti-CMV prophylaxis for D+/R- patients [5]. 90% of respondents used Valganciclovir for prophylaxis [5].

Leukopenia and neutropenia are the most clinically relevant hematological toxicities among the anticipated adverse drug reactions of antiviral agents used in anti-CMV prophylaxis therapies [6]. Recent studies have shown inconsistent reporting of hematological adverse events in trials comparing prophylactic and preemptive therapies, with discrepancies ranging from no reported differences to a significant disparity of up to 30% versus 3% [7–13]. A 2023 randomized clinical trial (RCT) comparing letermovir and valganciclovir for prophylaxis in high-risk KT recipients found a higher rate of severe leukopenia or neutropenia in the valganciclovir group (64.0% versus 26.0%) [14]. In case of valganciclovir, dose reduction carries a risk of developing drug-resistant CMV strains [15].

To address the gap in the literature regarding the burdens of CMV prophylaxis in KT recipients, LECOCYT, an observational multicenter French study, aimed to primarily investigate the differences in leukopenia and neutropenia grade 3 or 4, between KT recipients who received anti-CMV prophylaxis (D+/R-) compared to untreated patients (D-/R-) over a 6-month period post-transplant, and to evaluate the clinical and economic burdens (associated with hematological toxicities).

## Materials and Methods

### Study Design and Population

The LECOCYT study was a multicenter, prospective, longitudinal, observational cohort study designed to examine the characteristics and outcomes of two distinct groups. Approval was obtained from the local ethics committee with the registration number 2021-A01250-41. The first group (D+/R-), consisted of high-risk CMV transplant recipients who received antiviral prophylaxis. The second group (D-/R-) did not receive prophylaxis due to their lower risk of developing CMV-related complications, thus avoiding hematological toxicities related to prophylaxis. This second group served as a comparator to describe the clinical burden associated with antiviral prophylaxis in the first group. This study design isolated the specific outcomes associated with prophylaxis in high-risk transplant recipients (D+/R-) from the transplant procedure itself.

To assess the economic burden, comparative analyses were performed within the D+/R- group stratified into subgroups based on the presence or absence of neutropenia and leukopenia grade 3 or 4.

Twenty-two French kidney-transplant centers participated in the study; the list of centers is available in the online data Supplementary Material S1. We included patients 18 years or older at the time of KT, transplanted within 10 days prior to the inclusion visit from a seropositive or seronegative donor, and CMV seronegative at the time of the KT. Non-opposition to patient-level data collection was obtained. The study was registered with number ID RCB 2021-A01250-41 and conducted in accordance with the ethical principles of the Declaration of Helsinki.

The primary objective was to compare the difference of severe leukopenia/neutropenia episodes between patients who received the prophylaxis for CMV (D+/R-) and untreated patients (D-/R-) within the first 6 months post-transplantation. Secondary objectives included the clinical and economic (variable costs) burdens associated with these toxicities. Healthcare resources utilization and medical costs were measured in D+/R- patients and included the medical time required for the management of patients, the duration of hospital stay, the number of subsequent hospitalization and the use of outpatient consultations. Exploratory objectives aimed to describe the use of anti-CMV prophylaxis, clinical outcomes, comedication, and quality of life (QoL) among these patients.

### Data Collection

The health and economic burden, in the D+/R- group was analyzed based on the presence or absence of neutropenia and leukopenia. An electronic Case Report Form (eCRF) was used for data collection.

Regarding hospital costs, the PMSI (*Programme de Médicalisation des Systèmes d’Informations*) is a French hospital information system designed to provide a standardized, medicalized measure of healthcare activity. For inpatient stays, this measurement relies on a coding system for procedures and diagnoses, the GHM (*Groupe Homogène de Malades*). GHM is derived from the PMSI, categorizes hospital stays into groups that are homogeneous in terms of medical characteristics and resource utilization. This classification is based on both administrative data (e.g., sex, age, length of stay) and medical data (e.g., diagnoses, procedures performed, comorbidities). Hospital procedure costs, stratified by GHM, were obtained from the French ATIH (*Agence Technique de l'Information sur l'Hospitalisation*) website. Inpatient costs were determined by linking the GHM code recorded in the electronic Case Report Form (eCRF) to the corresponding cost from the ATIH database. The weighted average procedure cost was calculated based on the length of hospital stay documented in the eCRF. Notably, hospital costs for subsequent KT-related procedures are fully covered (100%) by the French health insurance system.

The following information was collected: demographic data; medical and surgical history; medication review; clinical results; laboratory analyses; healthcare resource utilization (HCRU); costs incurred, estimated from the perspective of the French health insurance. The KT procedure was excluded from the cost analysis as it is a fixed cost common to all patients.

Patient-Reported Outcomes (PROs) instruments for QoL assessment were administered in paper format, using the Renal Transplant Quality of Life (ReTransQoL) and the Short Form 36 (SF-36) questionnaires. The SF-36 includes eight scores derived from section questions, each normalized to a 0–100 scale, where higher scores mean better QoL. The RTQ, tailored for KT patients, also scores overall QoL, with higher scores representing better quality of life. QoL scores were obtained at the time of inclusion in the study and during subsequent follow-ups at D30, D90, and D180.

Exposure to anti-CMV prophylaxis therapy was defined as the initiation of antiviral treatment (valganciclovir, ganciclovir, valaciclovir or acyclovir) within the first 10 days post-KT in patients who had no detectable CMV viremia at the time of the transplant and without evidence of active infection, indicating that these medications were prescribed solely for the purpose of preventing a potential CMV infection rather than treating an existing one. Follow-up visits for KT recipients were scheduled at approximately 30 days (D30), 90 days (D90), and 180 days (D180) post-transplant, following the standard of care in France. The occurrence of severe leukopenia or neutropenia was classified according to the Common Terminology Criteria for Adverse Events (CTCAE) version 5.0.

Grades of “White Blood Cell (WBC) count decreased” (i.e., leukopenia) are defined as follows:

- Grade 3: total WBC between 2,000 and 1,000/mm^3^ (or 2.0–1.0 × 10^9^/L)
- Grade 4: total WBC <1,000/mm^3^ (or <1.0 × 10^9^/L)

Grades of “neutrophil count decreased” (i.e., neutropenia) are defined as follows:

- Grade 3: Absolute Neutrophil Count (ANC) between 1,000 and 500/mm^3^ (or 1.0–0.5 × 10^9^/L)
- Grade 4: ANC <500/mm^3^ (or <0.5 × 10^9^/L)

Graft rejection was assessed using the Banff diagnostic classification [16], and death-censored graft loss was defined as the complete loss of kidney function post-transplant, which required chronic dialysis or retransplantation.

The medical time required for patient management estimated by the physician according to usual clinical practice, as reported on a visual analog scale (VAS) from 0 to 10, was collected at each follow-up visits, with a lower score indicating less time needed for medical management. The Visual Analog Scale (VAS) is a straightforward and validated tool commonly used to assess characteristics or attitudes that are thought to exist on a continuous scale but are difficult to measure directly. Thus, it can be used to gauge a physician’s perception of the time spent managing a patient in a real-world setting.

### Statistical Analysis

Categorical variables were presented as frequencies and proportions, and continuous data were presented by mean, standard deviation (SD), median, minimum, maximum, and quartiles (Q1 and Q3).

The primary objective of this study was analyzed using a multivariate logistic regression model to compare the incidence of hematological toxicities and potential associated factors in the D-/R- group versus the D+/R- group by calculating OR with 95% CI. Univariate logistic regression was employed to assess the potential impact of each covariate with a p-value of ≤0.2 for inclusion in the multivariable regression. The final multivariable regression model included age, sex, mycophenolic acid, corticosteroids, absence of certain co-medications (mTOR inhibitors and sulfamethoxazole/trimethoprim), proton pump inhibitors, and investigator sites. For numerical data related to secondary or exploratory objectives, t-test or Mann-Whitney U test was used, depending on distribution and sample size. A linear mixed model was used for repeated measurements. The type 1 error rate for establishing significance was set at 0.05. To avoid unnecessary multiple comparisons, no statistical tests were applied to descriptive outcomes such as clinical profile of patients (Table 5). In order to mitigate the risk of false positive a hierarchical approach of outcomes was followed for the analysis. All statistical analyses were performed in SAS (version 9.4) software.

## Results

### Patients

**FIGURE 1:**
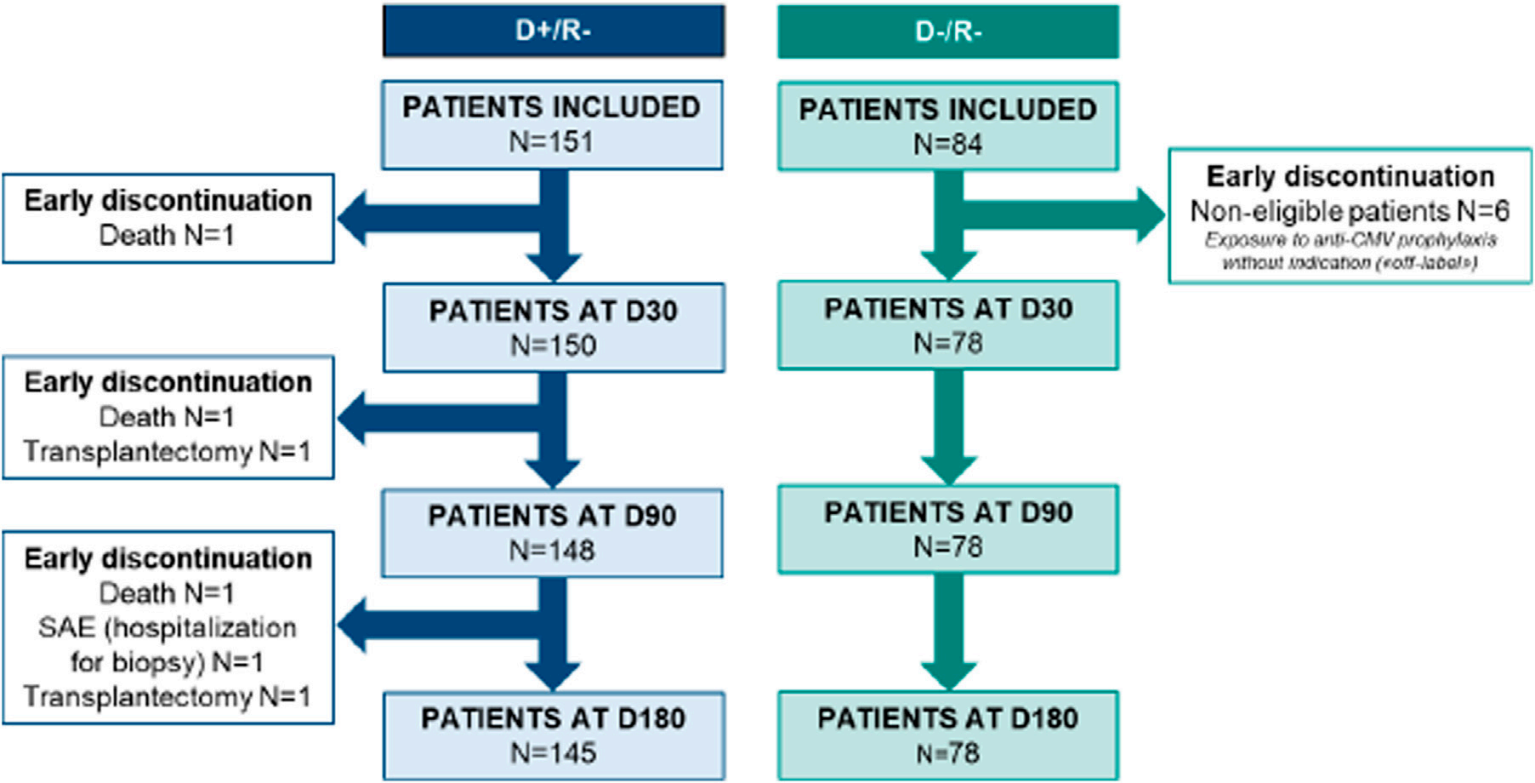
Flow chart of inclusion and follow-up of the study.

Between 3 September 2021, and 9 September 2022, a total of 235 patients were enrolled with enrollment ranging from 2 to 31 patients per center. Six patients were excluded from the D-/R- group due to their exposure to off-label anti-CMV prophylaxis. Finally, 229 patients were included in the analyzable population, with 151 from the D+/R- group and 78 from the D-/R- group. There were six early terminations in the D+/R- group before the end of the study: three patients died (one in each of the periods D0–D30, D30–D90, and D90–D180, respectively), two underwent transplantectomy with no renal graft function (one in each of the periods D30–D90 and D90–D180, respectively), and one experienced a serious adverse event, which was hospitalization for a biopsy (between D90 and D180) (Figure 1). 162/229 (70.9%) of the patients were male. The mean age of the patients was 57.2 ± 14.6 years. In the D+/R- group all patients received prophylaxis in the first 10 days post-KT, among them 91.4% (n = 138/151) received valganciclovir, 7.2% (n = 11/151) received ganciclovir and then switched to the oral form, and 2 patients (1.3%) were treated with valaciclovir (Table 1).

**TABLE 1:** Main features of the 229 study patients, including 151 CMV-seropositive donors (D+/R-) and 78 CMV-seronegative donors (D-/R-).

Variable	Patients D+/R- (n = 151)	Patients D-/R- (n = 78)	Total (N = 229)
Male, n (%)	110 (72.8)	52 (66.7)	162 (70.7)
Recipient age (years), mean (sd)	58.7 (14.9)	54.1 (13.8)	57.2 (14.6)
Body mass index, kg/m^2^, mean (sd), n = 225	26.3 (4.6)	25.2 (4.7)	26.0 (4.7)
Cardiovascular disease, n (%)	29 (19.2)	16 (20.5)	45 (19.7)
Chronic obstructive pulmonary disease, n (%)	9 (6.0)	2 (2.6)	11 (4.8)
Peptic ulcer disease, n (%)	6 (4.0)	2 (2.6)	8 (3.5)
Liver disease, n (%)	2 (1.3)	1 (1.3)	3 (1.3)
Diabetes mellitus, n (%)	24 (15.9)	15 (19.2)	39 (17.0)
Solid tumor, n (%)	14 (9.3)	8 (10.3)	22 (9.6)
Dialysis history, n (%)	126 (83.4)	67 (85.9)	193 (84.3)
Duration of dialysis (months), mean (sd), n = 193	35.1 (28.5)	31.8 (22.6)	34.0 (26.6)
Pre-emptive kidney transplant, n (%)	25 (16.6)	11 (14.1)	36 (15.7)
Rank of kidney transplant			
Transplant rank equal to 1, n (%)	134 (88.7)	65 (83.3)	199 (86.9)
Transplant rank equal to 2, n (%)	15 (9.9)	11 (14.1)	26 (11.4)
Transplant rank greater than 2, n (%)	2 (1.3)	2 (2.6)	4 (1.7)
Anti-CMV prophylaxis treatment			
Valganciclovir, n (%)	138 (91.4)	-	138 (60.3)
Ganciclovir, n (%)	11 (7.3)	-	11 (4.8)
Valaciclovir, n (%)	2 (1.3)	-	0 (0.0)
Time from KT to prophylaxis initiation (days), mean (sd), n = 150	3.6 (2.7)	-	3.6 (2.7)
Time from CMV viremia analysis to transplant procedure (days), mean (sd), n = 89	2.2 (8.6)	0.9 (1.8)	1.7 (6.8)
Treatment with Sulfamethoxazole/ Trimethoprim, n (%)	92 (60.9)	48 (61.5)	140 (61.1)
Immunosuppressive drugs at baseline			
Polyclonal antibodies (n, %)	41 (27.2)	21 (26.9)	62 (27.1)
Rituximab (n, %)	1 (0.7)	0 (0.0)	1 (0.4)
Basiliximab (n, %)	81 (53.6)	39 (50.0)	120 (52.4)
Belatacept (n, %)	0 (0.0)	0 (0.0)	0 (0.0)
Azathioprine (n, %)	1 (0.7)	0 (0.0)	1 (0.4)
Mycophenolic acid (n, %)	133 (88.1)	71 (91.0)	204 (89.1)
Cyclosporine (n, %)	9 (6.0)	4 (5.1)	13 (5.7)
Tacrolimus (n, %)	133 (88.1)	69 (88.5)	202 (88.2)
Everolimus (n, %)	7 (4.6)	1 (1.3)	8 (3.5)
Corticosteroids (n, %)	135 (89.4)	71 (91.0)	206 (90.0)
Eculizumab (n, %)	0 (0.0)	1 (100.0)	1 (100.0)

Abbreviations: sd, standard deviation; CMV, cytomegalovirus; KT, kidney transplantation; D+/R-, CMV-seropositive donors/CMV-seronegative recipients; D-/R-, CMV-seronegative donors/CMV, seronegative recipient; cardiovascular disease includes myocardium infarction, congestive heart failure, peripheral vascular disease and cerebrovascular accident or transient ischemic attack.

### Leukopenia and Neutropenia Episodes

In the D+/R– group, 26.5% of patients (n = 40/151) experienced at least one episode of leukopenia or neutropenia grade 3 or 4, compared to 7.7% of patients (n = 6/78) in the D-/R- group over a 6-month follow-up period. The odds ratio (OR) calculated using univariate logistic regression was 4.32 [95% confidence interval: 1.74–10.72], after adjustment with the multivariate model, OR was 5.16 [95% CI: 1.97–13.53] indicating that patients in the D+/R- group who received anti-CMV prophylaxis were over five times more likely to experience at least one episode of severe leukopenia/neutropenia compared to those in the D-/R- group (Table 2).

**TABLE 2:** Incidence of grade 3 or 4 leukopenia and neutropenia in patients during a 6-month follow-up period.

Variable	Patients D+/R– (n = 151)	Patients^a^ D–/R– (n = 78)	Unadjusted Odds Ratio (OR) [95% Confidence Interval (CI)]	Adjusted Odds Ratio (OR)^b^ [95% Confidence Interval (CI)]
Leukopenia or neutropenia Grade 3 or 4 (n, %)	40 (26.5)	6 (7.7)	4.32 [1.74–10.72]	5.16 [1.97–13.53]

^a^ D-/R- is considered as the reference group for the odds ratio (logistic regression).

^b^ Covariates: age, sex, mycophenolic acid, corticosteroids, absence of certain co-medications (mTOR, inhibitors and sulfamethoxazole/trimethoprim), proton pump inhibitors, and investigator sites.

A total of 79 episodes of leukopenia or neutropenia grade 3 or 4 were recorded in 46 patients, 71 episodes in the D+/R- group (n = 40 patients) and 8 episodes in the D-/R- group (n = 6 patients). The average duration of these episodes was 16.2 ± 15.8 days with their onset typically occurring around 3 months post-transplant, at a mean of 3.0 ± 1.4 months (Table 3).

**TABLE 3:** Description of grade 3 or 4 leukopenia or neutropenia in patients during a 6-month follow-up period.

Variable	Patients D+/R- (n = 151)	Patients D-/R- (n = 78)	Total (N = 229)
Leukopenia or neutropenia Grade 3 or 4 (n, %)	40 (26.5)	6 (7.7)	46 (20.1)
Number of leukopenia or neutropenia episodes of grade 3 or 4, n	71	8	79
Duration of leukopenia or neutropenia episodes of grade 3 or 4 (days), mean (sd)	15.5 (15.6)	21.6 (17.0)	16.2 (15.8)
Time of diagnosis since KT procedure (months), mean (sd)	3.0 (1.4)	3.2 (1.3)	3.0 (1.4)

Abbreviations: sd, standard deviation; KT, kidney transplantation; D+/R-, seropositive donors/seronegative recipients for cytomegalovirus; D-/R-, seronegative donors/seronegative recipients for cytomegalovirus.

In the D-/R- group, 1 episode of grade 4 leukopenia and 3 episodes (for 3 patients) of grade 3 leukopenia were declared. Regarding neutropenia in the same group, 3 episodes (for 3 patients) of grade 4 and 1 episode of grade 3 was declared.

In the D+/R-, 7 episodes (for 7 patients) of grade 4 leukopenia and 29 episodes of grade 3 leukopenia (24 patients had one episode, and 1 patient had 5 episodes) were declared. Regarding neutropenia in the same group, 13 episodes (11 patients had one episode and 1 patient had 2 episodes) of grade 4 and 22 episodes (for 22 patients) of grade 3 were declared.

**FIGURE 2:**
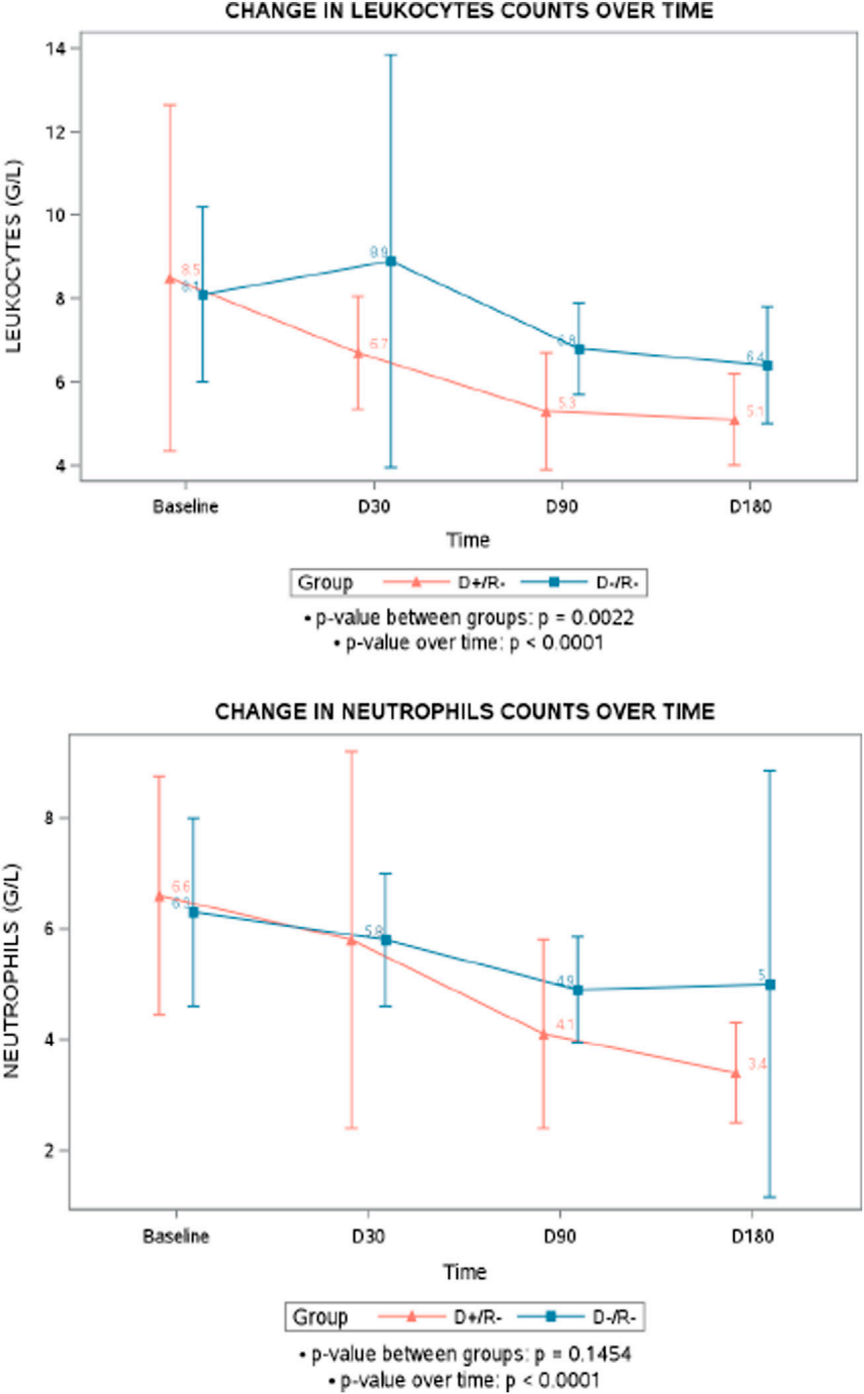
Mean change in leukocytes and neutrophils counts over time. D+/R-, seropositive donors/ seronegative recipients for cytomegalovirus; D-/R-, seronegative donors/ seronegative recipients for cytomegalovirus.

Besides the occurrence of neutropenia or leukopenia, a general trend of decreasing counts of leukocyte and neutrophil after transplantation was observed over time (p < 0.0001), with the D+/R- group showing a more pronounced decline in leukocytes compared to the D-/R- group (p = 0.0022). However, while the neutrophil counts significantly decreased over time in the D+/R- (p < 0.0001), the difference between D+/R- and D-/R- was not statistically significant (p = 0.1454) (Figure 2).

The use of at least one dose of G-CSF occurred in 16 patients (10.6%) in D+/R- group versus 4 patients (5.1%) in the D-/R- group.

In the Lecocyt study, 16 patients in the D+/R- group experienced a CMV disease episode during the 6-month follow-up period, with a mean time from KT procedure to diagnosis of 107.8 days (SD: 50.4 days) and a median of 122.5 days. In the D-/R- group, only one event was reported, with diagnosis occurring 109 days after the procedure. Regarding grade 3 or 4 neutropenia/leukopenia episodes, the mean time from the procedure to diagnosis was 91.9 days (SD: 42.1) with a median of 92 days for the D+/R- group and a mean time of 95.9 days (SD: 40.7) with a median of 109 days for the D-/R- group. In general, especially in the D+/R-, the episodes of neutropenia/leukopenia occurred before the episode of CMV disease.

### Utilization Pattern of Current Anti–CMV Medication

Our study found that all D+/R- patients received prophylactic treatment within the first 10 days post-transplant. In this group, 138/151 patients (91.4%) initiated prophylaxis with valganciclovir, indicating its widespread adoption in clinical practice (Table 4). Ganciclovir was the initial prophylactic treatment administered to 11/151 patients (7.3%), all of whom subsequently switched to valganciclovir. The specific dosages of valganciclovir and ganciclovir are detailed in Table 4. The remaining 2/151 patients (1.3%) received only valaciclovir as their initial prophylactic treatment throughout the study (Table 4).

**TABLE 4:** Description of utilization pattern of anti–CMV medication regimen within the 6–months post–KT.

Variable	Patients D+/R- (n = 151 patients)
Patients with anti-CMV prophylaxis treatment at baseline	151 (100.0)
Valganciclovir (%)	138 (91.4)
Ganciclovir (%)	11 (7.3)
Valaciclovir (%)	2 (1.3)
Dosage of valganciclovir at baseline (mg/day)	
Number of patients, n	138^a^
<225, n (%)	29 (21.0)
225, n (%)	29 (21.0)
450, n (%)	73 (52.9)
750, n (%)	1 (0.7)
900, n (%)	24 (17.4)
Dosage of ganciclovir at baseline (mg/day)	
Number of patients, n	11^a^
<100, n (%)	5 (45.5)
100–200, n (%)	9 (81.8)
>200, n (%)	5 (45.5)
Valganciclovir administered during the study	149
Duration of continuous treatment administration (days), mean (sd), n = 113 patients	160.8 (44.0)
*Reasons for permanent end of treatment before the 6-month follow-up (n = 50 patients)*	
Planned end of treatment, n (%)	34 (68.0)
Permanent interruptions due to hematological toxicities (leukopenia or neutropenia, any grade), n (%)	12 (24.0)
Permanent interruptions due to resistance to treatment, n (%)	3 (6)
Permanent interruptions due to cholestasis, n (%)	1 (2.0)
Total number of days of treatment exposure with temporary interruptions^b^, mean (sd), n = 36 patients	110.8 (47.5)
Duration of treatment temporary interruptions (number of days of non-exposure), mean (sd), n = 42 interruptions	20.3 (26.5)
Temporary interruptions due to hematological toxicities, n (%)	12 (33.3)
Ganciclovir administered during the study	15
Duration of continuous treatment administration (days), mean (sd), n = 3 patients	8.0 (4.6)
Permanent interruptions due to resistance to treatment, n (%)	1 (33.3)
Total number of days of treatment exposure with temporary interruptions^b^, mean (sd), n = 12 patients	12.8 (9.9)
Duration of temporary treatment interruptions (number of days of non- exposure), mean (sd), n = 14 interruptions	93.5 (130.8)

^a^ Several patients received different doses of treatment.

^b^ Number of days of exposure excluding the time of interruption.

Abbreviations: sd, standard deviation; CMV, cytomegalovirus; KT, kidney transplantation; D+/R-, CMV-seropositive donors/CMV-seronegative recipients; D-/R-, CMV-seronegative donors/CMV-seronegative recipients.

During the 6-month follow-up period, 113 out of 149 D+/R- patients (75.8%) who were administered valganciclovir, received continuous treatment without any interruption recorded, with a mean duration of exposure of 160.8 ± 44.0 days. Out of these 113 patients, 63 (55.8%) continued valganciclovir until the end of the study. Permanent treatment interruptions were recorded for 50/113 patients (44.2%) before the end of the 6-month follow-up period, with the primary reasons being the planned end of treatment (n = 34, 68.0%) and hematological toxicities (n = 12, 24.0%). Temporary valganciclovir treatment interruptions were observed in 36/149 patients (24.2%), including 12/36 patients (33.3%) for hematological toxicity (Table 4).

During the 6-month follow-up period, 15 out of 149 D+/R- patients (10.1%) were treated with ganciclovir. Temporary treatment interruptions were reported in 12 patients (Table 4).

### Clinical Follow-Up

Among the total of 229 patients, 39 (17%) experienced at least one episode of infection requiring hospitalization during the 6-month follow-up period: 30 (19.9%) in the D+/R- group and 9 (11.5%) in the D-/R- group. Graft rejection was experienced by 10 patients (6.6%) in the D+/R- group and 3 (3.8%) in the D-/R- group. Of these, graft loss occurred in 7 patients (4.6%) of the D+/R- group and 2 (2.6%) of the D-/R- group, respectively (Table 5).

**TABLE 5:** Clinical outcomes and infectious complications in KT Recipients within the 6–months post–KT.

Variable	Patients D+/R– (n = 151 patients)	Patients D–/R– (n = 78 patients)	Total (N = 229 patients)
Patients with at least one episode of infection requiring hospitalization^a^, n (%)	30 (19.9)	9 (11.5)	39 (17.0)
Episodes of infection requiring hospitalization per patient, mean (sd), n = 50 episodes, n = 39	1.3 (0.6)	1.1 (0.3)	1.3 (0.6)
Patients with one episode of zona, n (%)	1 (0.7)	1 (1.3)	2 (0.9)
Patients with graft rejection, n (%)	10 (6.6)	3 (3.8)	13 (5.7)
Death-censored graft loss, n (%)	7 (4.6)	2 (2.6)	9 (3.9)
Patient with an episode of CMV disease during the 6-month period, n	16	1	17
Time between KT procedure and diagnostic of the episode, mean (sd), n = 17	107.8 (50.4)	109.0 (.)	107.8 (48.8)
Patients requiring hospitalization due to an episode of CMV Infection, n (%)	8 (50.0)	0 (0.0)	8 (47.1)
Death, n (%)	3 (2.0)	0 (0.0)	3 (1.3)
Causes of death			
Sudden and unexpected death, n (%)	2 (66.7)	0 (0.0)	2 (66.7)
Subdural hematoma due to a fall, n (%)	1 (33.3)	0 (0.0)	1 (33.3)

^a^ Infections other than CMV (e.g., viral, bacterial, parasitic, etc.) requiring hospitalization.

Abbreviations: sd, standard deviation; CMV, cytomegalovirus; KT, kidney transplantation; D+/R-, CMV-seropositive donors/CMV-seronegative recipients; D-/R-, CMV-seronegative donors/CMV-seronegative recipients.

Out of 229 patients, 17 (7.4%) experienced an episode of symptomatic CMV disease (which was detected at the discretion of the physician), with 16 (94.1%) of these patients being in the D+/R- group and only one patient being in the D-/R- group. The average time between KT and the diagnosis of the episode of CMV disease was 107.8 ± 48.8 days. Hospitalization due to CMV infection was required in 8 (47.1%) patients, all of whom were in the D+/R- group. In total, three deaths occurred during the study period, all within the D+/R- group. None of the deaths were related to CMV-disease or CMV-prophylaxis.

### Quality of Life

The SF-36 results showed an overall significant improvement in QoL over 6 months post-transplant, with no significant differences between D+/R- and D-/R- groups, except in social functioning (p = 0.0282), where the D-/R- group had a better outcome. Additionally, the RTQ total score demonstrated both an overall significant improvement in QoL over the same period and also a significant difference between the two groups (p = 0.0048), with the D-/R- group reporting a higher mean score of 75.2 (10.7) compared to 71.3 (10.6) for D+/R- group at 6 months, reflecting a better QoL (Table 6). Details on QoL scores are available in the online data Supplementary Material S2.

**TABLE 6:** Analysis of QoL over time between D+/R- and D-/R- groups using SF-36 and RTQ questionnaires.

Variable	p-value of significance between the two groups^a^ D + R- (n = 151) and D-R- (n = 78)	p-value of significance over time^a^ Inclusion, D30, D90 and D180
SF-36		
Physical functioning	0.7307	<0.0001
Role limitations due to physical health	0.1808	<0.0001
Role limitations due to emotional problems	0.4930	0.0080
Energy/fatigue	0.4025	0.0024
Emotional wellbeing	0.2708	0.0085
Social functioning	0.0282	0.0032
Pain	0.5841	0.0334
General health	0.6258	0.0991
RTQ		
RTQ total score	0.0048	0.0123
Physical Health	0.0411	<0.0001
Social Functioning	0.1846	0.7980
Medical care and satisfaction	0.0364	0.0746
Treatment	0.0219	0.9777
Fear and loosing graft	0.1153	0.0013

^a^ Mixed model for repeated measures.

Abbreviations: D+/R-, seropositive donors/seronegative recipients for cytomegalovirus; D-/R-, seronegative donors/seronegative recipients for cytomegalovirus; D30, day 30; D90, day 90; D180, day 180; SF-36, Short Form 36; RTQ, renal transplant quality of life.

### Healthcare and Economic Burden in D+/R- Patients

The subgroup of patients D+/R- who experienced at least one episode of neutropenia/leukopenia post-transplant (n = 40) incurred significantly higher medical costs for their medical follow-ups, with a median of approximately €4,500 (Q1 = €561; Q3 = €10,000), compared to the sub-group of those without any neutropenia/leukopenia (n = 111), which had a median cost of nearly €2,100 (Q1 = €182; Q3 = €6,500) (p = 0.02) (Table 7). Additionally, the subgroup of D+/R- patients with neutropenia/leukopenia had a higher number of subsequent hospitalizations following the transplant procedure (mean of 3.5 ± 3.1) compared to the subgroup of those without neutropenia/leukopenia (mean of 2.3 ± 2.0; p = 0.050); however, the duration of hospital stays did not differ significantly between the two subgroups. Furthermore, the subgroup of D+/R- patients with at least one episode of neutropenia/leukopenia had a higher mean number of outpatient consultations per patient post-transplant until D180 (5.5 ± 4.1 consultations) compared to the subgroup of D+/R- patients without episodes of neutropenia/leukopenia (4.5 ± 3.3 consultations; p = 0.042) (TABLE 7).

**TABLE 7:** Comparative analysis of post-transplant healthcare costs and utilization between D+/R- patients with and without neutropenia/leukopenia episodes.

Variable	Patients D+/R- with at least one episode of Neutropenia /leukopenia^a^ (n = 40)	Patients D+/R- with no episode of Neutropenia /leukopenia^a^ (n = 111)	Total (N = 151)	p^b^
Total post-transplant costs (EUR) (index procedure not included)				0.025
Number of patients	39	91	130	
Mean (SD)	7,593 (9,424)	4,456 (6,038)	5,397 (7,327)	
Median	4,515	2073	2,595	
Min – Max	99–35,720	17–25,183	17–35,720	
Inpatients costs (follow-up procedures) (EUR)				0.090
Number of patients	27	56	83	
Mean (SD)	10,395 (9,943)	6,890 (6,450)	8,030 (7,873)	
Median	6,961	4,488	5,399	
Min – Max	470–35,720	470–25,133	470–35,720	
Outpatient consultation costs (EUR)				0.091
Number of patients	35	80	115	
Mean (SD)	228 (163)	173 (141)	190 (149)	
Median	198	165	165	
Min – Max	17–611	17–594	17–611	
Length of stay in the service of admission of index procedure (in days)				0.683
Number of patients	40	110	150	
Mean (SD)	12.7 (5.5)	12.9 (6.3)	12.8 (6.1)	
Median	11.0	12.0	11.0	
Min – Max	5–28	4–54	4–54	
Number of subsequent hospitalizations				0.050
Number of patients	24	52	76	
Mean (SD)	3.5 (3.1)	2.3 (2.0)	2.6 (2.4)	
Median	2.5	2.0	2.0	
Min – Max	1–14	1–13	1–14	
Duration of subsequent hospitalizations (days)				0.871
Number of hospitalizations (missing data)	83 (0)	114 (4)	197 (4)	
Mean (SD)	6.7 (5.3)	8.0 (9.0)	7.4 (7.7)	
Median	6.0	5.0	5.0	
Min – Max	1–32	1–47	1–47	
Number of outpatient consultation(s) per patients until D180				0.042
Mean (SD)	5.5 (4.1)	4.5 (3.3)	4.8 (3.6)	
Median	5.0	4.0	4.0	
Min – Max	1–25	1–20	1–25	

^a^ With at least one/no episode of neutropenia/leukopenia grade 3 or 4 within the first 6 months post-KT.

^b^ Wilcoxon–Mann–Whitney test.

Abbreviations: sd, standard deviation; D+/R-, seropositive donors/seronegative recipients for cytomegalovirus; D-/R-, seronegative donors/seronegative recipients for cytomegalovirus; D180, day 180.

**FIGURE 3:**
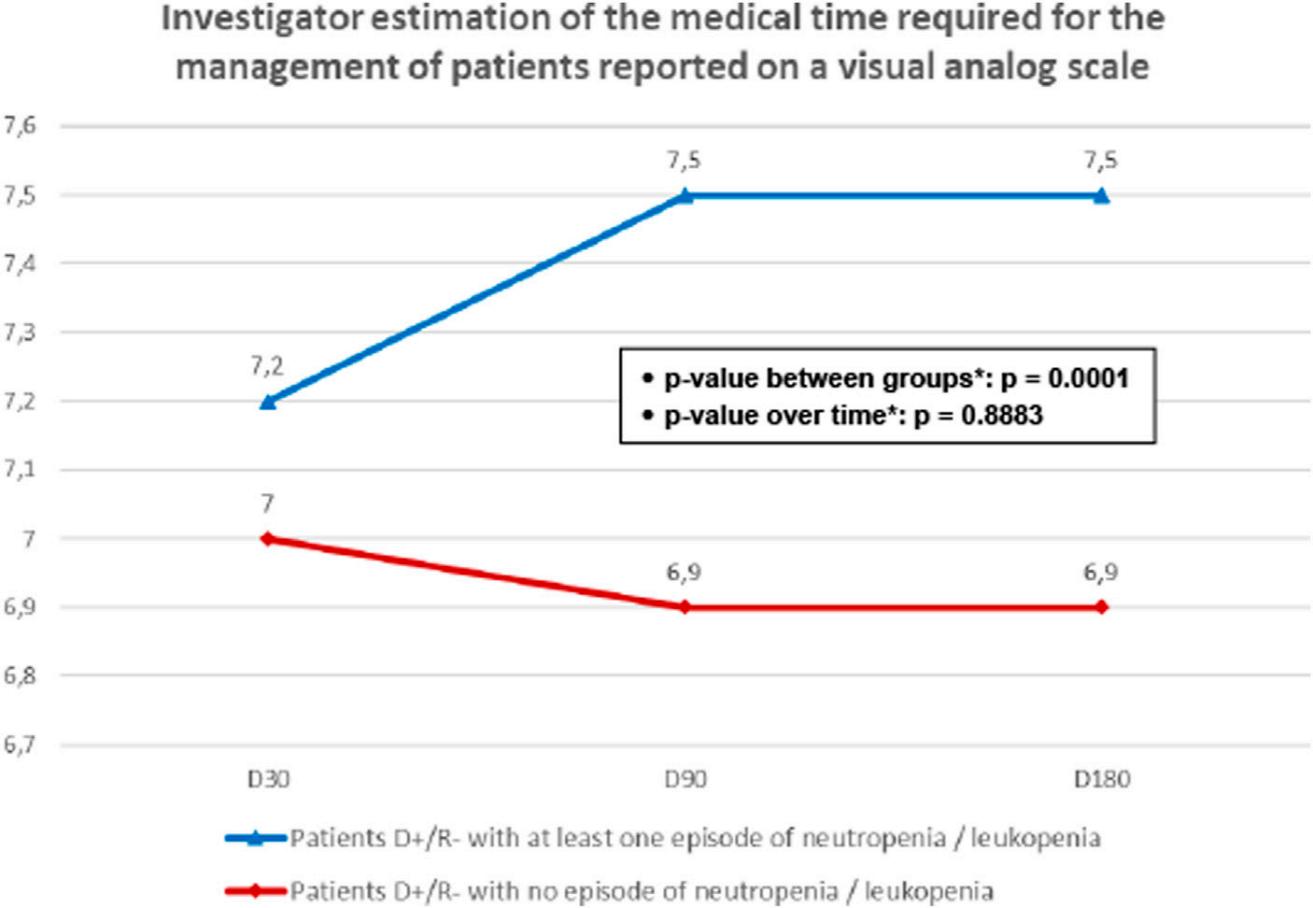
Investigator estimation of the medical time required for the management of patients reported on a visual analog scale. D+/R-, seropositive donors/ seronegative recipients for cytomegalovirus; D-/R-, seronegative donors/ seronegative recipients for cytomegalovirus. *Mixed model for repeated measures.

During the follow up period, patients with neutropenia/leukopenia required more medical time, as indicated by higher average scores on the VAS scale of 7.5 ± 1.1 at D90 and 7.5 ± 1.0 at D180, compared to scores of 6.9 ± 0.9 and 6.9 ± 1.1 at D90 and D180, respectively, for the subgroup without neutropenia/leukopenia. This difference was statistically significant (p = 0.0001), as shown in Figure 3.

## Discussion

In the LECOCYT study, the high-risk D+/R- group received anti-CMV prophylaxis, which was associated with a statistically significant increase in the incidence of severe leukopenia or neutropenia (grade 3 or 4) in the first 6 months following KT: 26.6% in the D+/R- group who received anti-CMV prophylaxis versus 7.7% in the untreated D-/R- group.

The adjusted OR for confounding factors was 5.16 [95% CI: 1.97–13.53], indicating that patients in the D+/R- group were more than five times as likely to develop severe leukopenia or neutropenia compared to the D-/R- group. This is likely related to the use of valganciclovir, which was the main prophylaxis treatment used by 98.7% of patients. The association between valganciclovir and higher rates of leukopenia or neutropenia is supported by a recent RCT and in a retrospective study that showed a higher incidence of leukopenia or neutropenia with valganciclovir compared to letermovir, a newer anti-CMV treatment, in adult D+/R- recipients over a 52-week period [14, 16]. Valganciclovir-based prophylaxis remains a widely used strategy for preventing CMV infection, but it has limitations beyond its hematologic toxicities such as the development of resistance or the need for close monitoring of kidney function for dose adjustments [14].

An alternative to prophylaxis is the preemptive therapy strategy, which requires a weekly monitoring of CMV DNAemia and the initiation of treatment when the latter is detected. This strategy may be responsible of less leucopenia. However, the risk of anti-CMV resistance is higher is D+/R- kidney-transplant patients receiving preemptive therapy [17]. Furthermore, in an international survey, it has been shown that most transplant physicians prefer prophylaxis to preemptive therapy in this setting [5].

Our study captured a difference in the time required for the management of patients of D+/R- with severe leukopenia or neutropenia despite a low number of outpatient consultations reported per patient post-transplant, as leukopenia/renal function is often managed remotely in current practice in France. The additional time required for the management of D+/R- with severe leukopenia or neutropenia may result from the necessity for dose adjustments based on renal function. D+/R- patients with severe neutropenia or leukopenia, scored higher on the VAS for medical time and required more rehospitalizations and outpatient consultations (p = 0.042) with higher total healthcare expenditures observed for these patients (p = 0.025), despite the variability and sample size limitations that warrant cautious interpretation of cost differentials. Increased healthcare costs post-transplant, associated with episodes of neutropenia or leukopenia have been reported [18, 19]. Our study is the first to assess the direct costs associated with severe leukopenia or neutropenia in D+/R- adults in France.

The study faced limitations in data collection, as actual costs were unavailable. Consequently, researchers used estimated costs based on standardized reimbursement package for hospital admissions, outpatient consultations and work absenteeism. Another limitation of our study is the exclusion of R+ patients, who typically receive prophylaxis for 3 months. Additionally, data on the dosages of mycophenolic acid (MPA) administered to patients was not available. MPA is an immunosuppressive agent commonly used in organ transplant recipients to prevent graft rejection. Accurate dosing information is crucial, as it can affect both the risk of infection and the incidence of hematological adverse events, including leukopenia and neutropenia. This study was funded by MSD France whom did not play any role in data collection and did not interfere in the results interpretation. The study was supervised by an internationally recognized expert committee with a strong track record in this field, ensuring rigorous oversight throughout the process. The study was carried out in strict adherence to all applicable clinical research standards and regulations, with full efforts to maintain the integrity of the research. Despite these constraints, the research offers important insights and is notable for being the only prospective, real-world study in France that explores the clinical and economic burden associated with hematological toxicities related to anti-CMV prophylaxis in D+/R- patients.

In conclusion, the LECOCYT study found that KT recipients receiving current anti-CMV prophylaxis (D+/R-) have a higher risk of severe hematologic toxicities compared to unexposed patients (D-/R-). D+/R patients with leukopenia or neutropenia grade 3 or 4 required more medical management time and incurred in higher costs than those without episodes.

## Data Availability

The datasets presented in this article are not readily available because of ethics restrictions. Requests to access the datasets should be directed to christophe.legendre@aphp.fr.

## Group Members of on behalf of the LECOCYT Study Group

Gabriel CHOUKROUN, CHU Amiens; Fatouma TOURE-DIABIRA, CHU Limoges; Laeticia ALBANO, Hôpital Pasteur 2; Mathilde LEMOINE, CHU Rouen; Alexandre HERTIG, Hôpital Foch; Thibault RENOUF, CHU Rennes; Antoine DURRBACH, Hôpital Henri-Mondor; Lionel ROSTAING, CHU Grenoble Alpes – Hôpital Nord Michallon; Claire TINEL, CHU Bocage, Antoine THIERRY, CHU Poitiers; Cyril GARROUSTE, CHRU Gabriel Montpied; Martin PLANCHAIS, CHU Angers; Peggy PERRIN, Nouvel Hôpital Civil Strasbourg, Magali GIRAL, CHU Nantes; Pierre MERVILLE, CHU Bordeaux; Yannick LE MEUR, CHRU Brest; Tristan LEGRIS, Hôpitaux Universitaires de Marseille Conception; Pierre GALICHON, La Pitié Salpêtrière; Betoul SCHVARTZ, CHU Reims; Philippe GATAULT, CHRU Tours; Christophe MARIAT, CHU Saint-Etienne – Hôpital Nord.

## Abbreviations

ANC, Absolute Neutrophil Count; ATIH, French Agency for Information on Hospital Care – Agence technique de l’information sur l’hospitalisation; CI, Confidence Interval; CMV, Cytomegalovirus; CTCAE, Common Terminology Criteria for Adverse Events; D-/R-, CMV-seronegative donors/CMV-seronegative recipients; D+/R-, CMV-seropositive donors/CMV-seronegative recipients; eCRF, Electronic Case Report Form; G-CSF, Granulocyte-Colony Stimulating Factor; GHM, Homogeneous group of patient’s classification – Groupe Homogène de Malades; HCRU, Healthcare Resource Utilization; KT, Kidney Transplant; MPA, Mycophenolic Acid; mTOR, Mechanistic Target of Rapamycin; OR, Odds Ratio; PMSI, French Information Systems Medicalization Program – Programme de Médicalisation des Systèmes d’Information; PROs, Patient-Reported Outcomes; Q1/Q3, Quartiles 1 and 3; QoL, Quality of Life; RCT, Randomized Clinical Trial; RTQ, Renal Transplant Quality of Life – ReTransQoL; SAS, Statistical Analysis System software; SD, Standard Deviation; SF-36, Short Form 36; VAS, Visual Analog Scale; WBC, White Blood Cell.
