# Supplementary material for: Clinical and Economic Burden Associated With Anti-Cytomegalovirus (CMV) Prophylaxis Therapies in Adult Kidney Transplant Recipients (LECOCYT): An Observational Study
Source: Transpl Int. 2025 May 19;38:14342. doi: 10.3389/ti.2025.14342 (PMC12127846; doi:10.3389/ti.2025.14342)
Supplement: Supplementary file 1 [file DataSheet1.docx]

Data supplement

Appendix S1

**List of participating centers and principal investigators**

|  | **PRINCIPAL INVESTIGATOR** | **CENTER** |
| --- | --- | --- |
| 1 | KAMAR Nassim | CHU Toulouse-Rangueil |
| 2 | CHOUKROUN Gabriel | CHU Amiens |
| 3 | TOURE – DIABIRA Fatouma | CHU Limoges |
| 4 | ALBANO Laetitia | Hôpital Pasteur 2 |
| 5 | LEMOINE Mathilde | CHU Rouen |
| 6 | MARIAT Christophe | CHU Saint-Etienne – Hôpital Nord |
| 7 | HERTIG Alexandre | Hôpital Foch |
| 8 | RENOUF Thibault | CHU Rennes |
| 9 | LEGENDRE Christophe | Hôpital Necker |
| 10 | DURRBACH Antoine | Hôpital Henri-Mondor |
| 11 | ROSTAING Lionel | CHU Grenoble Alpes – Hôpital Nord Michallon |
| 12 | TINEL Claire | CHU BOCAGE |
| 13 | THIERRY Antoine | CHU Poitiers |
| 14 | GARROUSTE Cyril | CHRU Gabriel Montpied |
| 15 | PLANCHAIS Martin | CHU Angers |
| 16 | PERRIN Peggy | Nouvel Hôpital Civil |
| 17 | GIRAL Magali | CHU Nantes |
| 18 | MERVILLE Pierre | CHU Bordeaux |
| 19 | LE MEUR Yannick | CHRU Brest |
| 20 | LEGRIS Tristan | Hôpitaux Universitaires de Marseille Conception |
| 21 | GALICHON Pierre | La Pitié Salpêtrière |
| 22 | SCHVARTZ Betoul | CHU Reims |
| 23 | GATAULT Philippe | CHRU Tours |

Appendix S2

**SF-36 - dimensions scores (N=229 patients)**

**Inclusion visit**

|  | **Patients D+/R**– **(n=151 patients)** | **Patients D**–**/R**– **(n=78 patients)** | **Total (N=229 patients)** |
| --- | --- | --- | --- |
| **Physical functionning** |  |  |  |
| Sample size | 129 | 73 | 202 |
| Mean (sd) | 52.9 (27.7) | 56.5 (30.9) | 54.2 (28.9) |
| Median | 55.0 | 60.0 | 55.0 |
| Q1 – Q3 | 35.0 - 75.0 | 30.0 - 80.0 | 35.0 - 80.0 |
| Min – Max | 0.0 - 100.0 | 0.0 - 100.0 | 0.0 - 100.0 |
| **Role limitations due to physical health** |  |  |  |
| Sample size | 124 | 70 | 194 |
| Mean (sd) | 42.6 (26.7) | 45.6 (25.1) | 43.7 (26.1) |
| Median | 42.7 | 50.0 | 43.8 |
| Q1 – Q3 | 25.0 - 56.3 | 25.0 - 62.5 | 25.0 - 62.5 |
| Min – Max | 0.0 - 100.0 | 0.0 - 100.0 | 0.0 - 100.0 |
| **Role limitations due to emotional problems** |  |  |  |
| Sample size | 123 | 70 | 193 |
| Mean (sd) | 58.6 (30.5) | 60.7 (31.8) | 59.4 (30.9) |
| Median | 58.3 | 58.3 | 58.3 |
| Q1 – Q3 | 33.3 - 83.3 | 33.3 - 91.7 | 33.3 - 83.3 |
| Min – Max | 0.0 - 100.0 | 0.0 - 100.0 | 0.0 - 100.0 |
| **Energy / fatigue** |  |  |  |
| Sample size | 126 | 72 | 198 |
| Mean (sd) | 47.5 (23.0) | 52.9 (23.8) | 49.4 (23.4) |
| Median | 46.9 | 56.3 | 50.0 |
| Q1 – Q3 | 31.3 - 62.5 | 31.3 - 71.9 | 31.3 - 68.8 |
| Min – Max | 0.0 - 100.0 | 0.0 - 93.8 | 0.0 - 100.0 |
| **Emotional well–being** |  |  |  |
| Sample size | 126 | 72 | 198 |
| Mean (sd) | 65.7 (21.4) | 67.8 (20.8) | 66.5 (21.1) |
| Median | 70.0 | 75.0 | 70.0 |
| Q1 – Q3 | 50.0 - 85.0 | 50.0 - 85.0 | 50.0 - 85.0 |
| Min – Max | 0.0 - 100.0 | 0.0 - 100.0 | 0.0 - 100.0 |
| **Social functioning** |  |  |  |
| Sample size | 126 | 73 | 199 |
| Mean (sd) | 64.5 (27.9) | 63.7 (28.9) | 64.2 (28.2) |
| Median | 62.5 | 62.5 | 62.5 |
| Q1 – Q3 | 50.0 - 87.5 | 37.5 - 87.5 | 50.0 - 87.5 |
| Min – Max | 0.0 - 100.0 | 0.0 - 100.0 | 0.0 - 100.0 |
| **Pain** |  |  |  |
| Sample size | 127 | 72 | 199 |
| Mean (sd) | 68.6 (25.9) | 66.3 (27.2) | 67.7 (26.3) |
| Median | 67.5 | 67.5 | 67.5 |
| Q1 – Q3 | 45.0 - 100.0 | 45.0 - 90.0 | 45.0 - 100.0 |
| Min – Max | 10.0 - 100.0 | 0.0 - 100.0 | 0.0 - 100.0 |
| **General health** |  |  |  |
| Sample size | 129 | 73 | 202 |
| Mean (sd) | 50.6 (17.7) | 53.9 (21.2) | 51.8 (19.0) |
| Median | 50.0 | 50.0 | 50.0 |
| Q1 – Q3 | 40.0 - 60.0 | 35.0 - 70.0 | 40.0 - 65.0 |
| Min – Max | 10.0 - 100.0 | 15.0 - 100.0 | 10.0 - 100.0 |

**Visit at D30**

|  | **Patients D+/R**– **(n=151 patients)** | **Patients D**–**/R**– **(n=78 patients)** | **Total (N=229 patients)** |
| --- | --- | --- | --- |
| **Physical functionning** |  |  |  |
| Sample size | 133 | 72 | 205 |
| Mean (sd) | 60.7 (25.0) | 61.2 (26.8) | 60.9 (25.6) |
| Median | 65.0 | 70.0 | 65.0 |
| Q1 – Q3 | 40.0 - 80.0 | 40.0 - 85.0 | 40.0 - 80.0 |
| Min – Max | 0.0 - 100.0 | 5.0 - 95.0 | 0.0 - 100.0 |
| **Role limitations due to physical health** |  |  |  |
| Sample size | 131 | 68 | 199 |
| Mean (sd) | 37.0 (24.4) | 43.0 (25.9) | 39.0 (25.0) |
| Median | 37.5 | 46.9 | 37.5 |
| Q1 – Q3 | 18.8 - 50.0 | 25.0 - 62.5 | 18.8 - 56.3 |
| Min – Max | 0.0 - 100.0 | 0.0 - 100.0 | 0.0 - 100.0 |
| **Role limitations due to emotional problems** |  |  |  |
| Sample size | 128 | 69 | 197 |
| Mean (sd) | 58.4 (29.3) | 57.4 (30.6) | 58.0 (29.7) |
| Median | 58.3 | 58.3 | 58.3 |
| Q1 – Q3 | 41.7 - 79.2 | 33.3 - 83.3 | 41.7 - 83.3 |
| Min – Max | 0.0 - 100.0 | 0.0 - 100.0 | 0.0 - 100.0 |
| **Energy / fatigue** |  |  |  |
| Sample size | 132 | 73 | 205 |
| Mean (sd) | 53.5 (21.2) | 54.3 (22.4) | 53.8 (21.6) |
| Median | 56.3 | 56.3 | 56.3 |
| Q1 – Q3 | 43.8 - 68.8 | 43.8 - 68.8 | 43.8 - 68.8 |
| Min – Max | 0.0 - 100.0 | 0.0 - 100.0 | 0.0 - 100.0 |
| **Emotional well–being** |  |  |  |
| Sample size | 132 | 73 | 205 |
| Mean (sd) | 72.4 (18.5) | 72.3 (20.7) | 72.4 (19.2) |
| Median | 75.0 | 80.0 | 75.0 |
| Q1 – Q3 | 60.0 - 85.0 | 65.0 - 85.0 | 60.0 - 85.0 |
| Min – Max | 10.0 - 100.0 | 5.0 - 100.0 | 5.0 - 100.0 |
| **Social functioning** |  |  |  |
| Sample size | 131 | 73 | 204 |
| Mean (sd) | 63.6 (26.6) | 62.3 (30.0) | 63.2 (27.8) |
| Median | 62.5 | 62.5 | 62.5 |
| Q1 – Q3 | 50.0 - 87.5 | 37.5 - 87.5 | 50.0 - 87.5 |
| Min – Max | 0.0 - 100.0 | 0.0 - 100.0 | 0.0 - 100.0 |
| **Pain** |  |  |  |
| Sample size | 132 | 70 | 202 |
| Mean (sd) | 70.5 (25.0) | 69.8 (27.0) | 70.2 (25.7) |
| Median | 77.5 | 68.8 | 77.5 |
| Q1 – Q3 | 45.0 - 95.0 | 55.0 - 90.0 | 45.0 - 90.0 |
| Min – Max | 10.0 - 100.0 | 0.0 - 100.0 | 0.0 - 100.0 |
| **General health** |  |  |  |
| Sample size | 133 | 73 | 206 |
| Mean (sd) | 56.0 (17.6) | 56.8 (18.5) | 56.3 (17.9) |
| Median | 55.0 | 55.0 | 55.0 |
| Q1 – Q3 | 45.0 - 65.0 | 40.0 - 70.0 | 45.0 - 70.0 |
| Min – Max | 10.0 - 95.0 | 15.0 - 95.0 | 10.0 - 95.0 |

**Visit at D90**

|  | **Patients D+/R**– **(n=151 patients)** | **Patients D**–**/R**– **(n=78 patients)** | **Total (N=229 patients)** |
| --- | --- | --- | --- |
| **Physical functionning** |  |  |  |
| Sample size | 125 | 69 | 194 |
| Mean (sd) | 72.7 (22.9) | 73.1 (24.4) | 72.8 (23.4) |
| Median | 80.0 | 80.0 | 80.0 |
| Q1 – Q3 | 60.0 - 90.0 | 65.0 - 90.0 | 60.0 - 90.0 |
| Min – Max | 0.0 - 100.0 | 15.0 - 100.0 | 0.0 - 100.0 |
| **Role limitations due to physical health** |  |  |  |
| Sample size | 123 | 68 | 191 |
| Mean (sd) | 53.6 (22.4) | 55.2 (27.0) | 54.2 (24.1) |
| Median | 56.3 | 50.0 | 50.0 |
| Q1 – Q3 | 37.5 - 68.8 | 37.5 - 75.0 | 37.5 - 68.8 |
| Min – Max | 0.0 - 100.0 | 0.0 - 100.0 | 0.0 - 100.0 |
| **Role limitations due to emotional problems** |  |  |  |
| Sample size | 122 | 68 | 190 |
| Mean (sd) | 65.3 (25.8) | 63.2 (29.6) | 64.6 (27.2) |
| Median | 66.7 | 66.7 | 66.7 |
| Q1 – Q3 | 50.0 - 83.3 | 41.7 - 95.8 | 50.0 - 91.7 |
| Min – Max | 0.0 - 100.0 | 0.0 - 100.0 | 0.0 - 100.0 |
| **Energy / fatigue** |  |  |  |
| Sample size | 126 | 68 | 194 |
| Mean (sd) | 56.5 (21.5) | 59.2 (25.5) | 57.4 (22.9) |
| Median | 56.3 | 59.4 | 56.3 |
| Q1 – Q3 | 37.5 - 75.0 | 43.8 - 78.1 | 43.8 - 75.0 |
| Min – Max | 0.0 - 100.0 | 0.0 - 100.0 | 0.0 - 100.0 |
| **Emotional well–being** |  |  |  |
| Sample size | 126 | 68 | 194 |
| Mean (sd) | 72.8 (19.2) | 72.9 (20.4) | 72.8 (19.6) |
| Median | 75.0 | 80.0 | 75.0 |
| Q1 – Q3 | 60.0 - 85.0 | 65.0 - 90.0 | 60.0 - 90.0 |
| Min – Max | 5.0 - 100.0 | 5.0 - 100.0 | 5.0 - 100.0 |
| **Social functioning** |  |  |  |
| Sample size | 126 | 69 | 195 |
| Mean (sd) | 71.8 (26.6) | 71.0 (26.9) | 71.5 (26.7) |
| Median | 75.0 | 75.0 | 75.0 |
| Q1 – Q3 | 50.0 - 100.0 | 50.0 - 100.0 | 50.0 - 100.0 |
| Min – Max | 0.0 - 100.0 | 0.0 - 100.0 | 0.0 - 100.0 |
| **Pain** |  |  |  |
| Sample size | 127 | 68 | 195 |
| Mean (sd) | 76.7 (23.4) | 77.4 (26.2) | 76.9 (24.3) |
| Median | 87.5 | 90.0 | 87.5 |
| Q1 – Q3 | 57.5 - 100.0 | 57.5 - 100.0 | 57.5 - 100.0 |
| Min – Max | 12.5 - 100.0 | 0.0 - 100.0 | 0.0 - 100.0 |
| **General health** |  |  |  |
| Sample size | 128 | 69 | 197 |
| Mean (sd) | 55.1 (18.8) | 59.2 (21.8) | 56.5 (20.0) |
| Median | 55.0 | 60.0 | 55.0 |
| Q1 – Q3 | 40.0 - 70.0 | 45.0 - 70.0 | 40.0 - 70.0 |
| Min – Max | 5.0 - 100.0 | 10.0 - 100.0 | 5.0 - 100.0 |

**Visit at D180**

|  | **Patients D+/R**– **(n=151 patients)** | **Patients D**–**/R**– **(n=78 patients)** | **Total (N=229 patients)** |
| --- | --- | --- | --- |
| **Physical functionning** |  |  |  |
| Sample size | 108 | 61 | 169 |
| Mean (sd) | 72.7 (22.2) | 80.1 (18.6) | 75.4 (21.2) |
| Median | 80.0 | 85.0 | 80.0 |
| Q1 – Q3 | 60.0 - 90.0 | 75.0 - 95.0 | 61.1 - 90.0 |
| Min – Max | 5.0 - 100.0 | 15.0 - 100.0 | 5.0 - 100.0 |
| **Role limitations due to physical health** |  |  |  |
| Sample size | 105 | 61 | 166 |
| Mean (sd) | 58.9 (25.9) | 65.1 (28.0) | 61.1 (26.8) |
| Median | 56.3 | 68.8 | 56.3 |
| Q1 – Q3 | 37.5 - 81.3 | 43.8 - 93.8 | 37.5 - 81.3 |
| Min – Max | 0.0 - 100.0 | 0.0 - 100.0 | 0.0 - 100.0 |
| **Role limitations due to emotional problems** |  |  |  |
| Sample size | 105 | 61 | 166 |
| Mean (sd) | 69.1 (27.2) | 73.2 (25.5) | 70.6 (26.6) |
| Median | 75.0 | 75.0 | 75.0 |
| Q1 – Q3 | 50.0 - 100.0 | 50.0 - 100.0 | 50.0 - 100.0 |
| Min – Max | 0.0 - 100.0 | 0.0 - 100.0 | 0.0 - 100.0 |
| **Energy / fatigue** |  |  |  |
| Sample size | 108 | 60 | 168 |
| Mean (sd) | 56.8 (20.3) | 65.8 (20.6) | 60.0 (20.8) |
| Median | 56.3 | 68.8 | 62.5 |
| Q1 – Q3 | 43.8 - 68.8 | 50.0 - 81.3 | 50.0 - 75.0 |
| Min – Max | 6.3 - 100.0 | 6.3 - 100.0 | 6.3 - 100.0 |
| **Emotional well–being** |  |  |  |
| Sample size | 108 | 60 | 168 |
| Mean (sd) | 72.3 (17.5) | 77.6 (16.4) | 74.2 (17.3) |
| Median | 75.0 | 80.0 | 80.0 |
| Q1 – Q3 | 60.0 - 85.0 | 70.0 - 85.0 | 65.0 - 85.0 |
| Min – Max | 20.0 - 100.0 | 25.0 - 100.0 | 20.0 - 100.0 |
| **Social functioning** |  |  |  |
| Sample size | 108 | 61 | 169 |
| Mean (sd) | 74.0 (24.4) | 78.7 (22.7) | 75.7 (23.9) |
| Median | 75.0 | 87.5 | 87.5 |
| Q1 – Q3 | 50.0 - 100.0 | 62.5 - 100.0 | 62.5 - 100.0 |
| Min – Max | 0.0 - 100.0 | 12.5 - 100.0 | 0.0 - 100.0 |
| **Pain** |  |  |  |
| Sample size | 106 | 61 | 167 |
| Mean (sd) | 75.1 (25.1) | 81.2 (19.5) | 77.4 (23.4) |
| Median | 77.5 | 90.0 | 80.0 |
| Q1 – Q3 | 57.5 - 100.0 | 65.0 - 100.0 | 57.5 - 100.0 |
| Min – Max | 0.0 - 100.0 | 35.0 - 100.0 | 0.0 - 100.0 |
| **General health** |  |  |  |
| Sample size | 108 | 61 | 169 |
| Mean (sd) | 54.0 (20.0) | 62.3 (21.1) | 57.0 (20.7) |
| Median | 55.0 | 65.0 | 55.0 |
| Q1 – Q3 | 40.0 - 70.0 | 50.0 - 75.0 | 40.0 - 75.0 |
| Min – Max | 10.0 - 95.0 | 5.0 - 100.0 | 5.0 - 100.0 |

Abbreviations: SF-36, Short Form 36; sd, standard deviation; Q1, first quartile; Q3, third quartile, D+/R-, seropositive donors/ seronegative recipients for cytomegalovirus; D-/R-, seronegative donors/ seronegative recipients for cytomegalovirus; D30, day 30; D90, day 90; D180, day 180.

**RetransQoL - global score (N=229 patients)**

**Inclusion visit**

|  | **Patients D+/R**– **(n=151 patients)** | **Patients D**–**/R**– **(n=78 patients)** | **Total (N=229 patients)** |
| --- | --- | --- | --- |
| **RTQ total score** |  |  |  |
| Number of patients | 130 | 73 | 203 |
| Mean (SD) | 68.9 (10.6) | 71.2 (9.3) | 69.7 (10.2) |
| Median | 69.6 | 71.3 | 70.2 |
| Q1 – Q3 | 62.4 – 77.1 | 65.1 – 77.7 | 63.3 – 77.3 |
| Min – Max | 38.2 – 89.9 | 48.5 – 90.2 | 38.2 – 90.2 |

**Visit at D30**

|  | **Patients D+/R**– **(n=151 patients)** | **Patients D**–**/R**– **(n=78 patients)** | **Total (N=229 patients)** |
| --- | --- | --- | --- |
| **RTQ total score** |  |  |  |
| Number of patients | 133 | 72 | 205 |
| Mean (SD) | 69.8 (10.3) | 71.5 (12.0) | 70.4 (11.0) |
| Median | 70.5 | 73.5 | 71.2 |
| Q1 – Q3 | 62.4 – 77.5 | 65.0 – 79.6 | 63.1 – 78.2 |
| Min – Max | 42.2 – 91.5 | 37.9 – 94.3 | 37.9 – 94.3 |

**Visit at D90**

|  | **Patients D+/R**– **(n=151 patients)** | **Patients D**–**/R**– **(n=78 patients)** | **Total (N=229 patients)** |
| --- | --- | --- | --- |
| **RTQ total score** |  |  |  |
| Number of patients | 128 | 69 | 197 |
| Mean (SD) | 71.9 (10.6) | 73.4 (12.5) | 72.4 (11.3) |
| Median | 71.8 | 73.7 | 72.5 |
| Q1 – Q3 | 66.1 – 80.2 | 65.8 – 82.1 | 66.0 – 80.6 |
| Min – Max | 37.8 – 91.2 | 33.6 – 95.5 | 33.6 – 95.5 |

**Visit at D180**

|  | **Patients D+/R**– **(n=151 patients)** | **Patients D**–**/R**– **(n=78 patients)** | **Total (N=229 patients)** |
| --- | --- | --- | --- |
| **RTQ total score** |  |  |  |
| Number of patients | 108 | 61 | 169 |
| Mean (SD) | 71.3 (10.6) | 75.2 (10.7) | 72.7 (10.7) |
| Median | 72.0 | 76.2 | 73.5 |
| Q1 – Q3 | 65.5 – 78.6 | 68.8 – 82.9 | 67.0 – 80.0 |
| Min – Max | 39.8 – 95.4 | 46.1 – 97.8 | 39.8 – 97.8 |

Abbreviations: RTQ, Renal Transplant Quality of Life; sd, standard deviation; Q1, first quartile; Q3, third quartile, D+/R-, seropositive donors/ seronegative recipients for cytomegalovirus; D-/R-, seronegative donors/ seronegative recipients for cytomegalovirus; D30, day 30; D90, day 90; D180, day 180
